# Trends in Group A *Streptococcus* Pharyngitis and Co-Infection with Severe Acute Respiratory Syndrome Coronavirus 2: A Retrospective Observational Study

**DOI:** 10.3390/medicina61050937

**Published:** 2025-05-21

**Authors:** Hidenori Takahashi, Yugo Satake, Saori Shimizu, Satomi Fujihara, Syunsuke Takano, Suzuko Fukasawa, Kaeyong Park, Naoya Toba, Takahiko Yano, Hiroki Nagamatsu, Ryutaro Hirose, Mio Toyama-Kousaka, Shinichiro Ota, Miwa Morikawa, Masaharu Shinkai

**Affiliations:** 1 Department of Respiratory Medicine, Tokyo Shinagawa Hospital, Tokyo 140-8522, Japanntoba634@gmail.com (N.T.); shin.ohta0915@gmail.com (S.O.);; 2 Department of Infection Control, Tokyo Shinagawa Hospital, Tokyo 140-8522, Japan; saokawachi@gmail.com (S.S.); fukasawa@tokyo-shinagawa.jp (S.F.); kansen@tokyo-shinagawa.jp (K.P.);

**Keywords:** group A *Streptococcus*, GAS pharyngitis, SARS-CoV-2, co-infection, rapid antigen test

## Abstract

*Background and Objectives*: Group A *Streptococcus* (GAS) is a leading cause of acute pharyngitis with seasonal outbreaks. The coronavirus disease 2019 (COVID-19) pandemic significantly altered respiratory infection trends; however, its impact on GAS pharyngitis (GAS-P) incidence remains unclear. Additionally, data on co-infections with GAS and severe acute respiratory syndrome coronavirus 2 (SARS-CoV-2) are limited. In this study, temporal trends in GAS-P incidence and characteristics of GAS–SARS-CoV-2 co-infections in Japan were examined. *Materials and Methods*: In this observational study, data from patients who visited the Tokyo Shinagawa Hospital between January 2019 and December 2024 were retrospectively analyzed. Data on GAS and SARS-CoV-2 test results and patient demographics were extracted from medical records. The study period was categorized based on COVID-19-related public health measures as follows: pre-COVID-19 social period (January 2019–April 2020), restricted social period (May 2020–April 2023), and post-restriction period (May 2023–December 2024). GAS incidence stratified by sex, age, and period was calculated. Clinical characteristics of patients co-infected with GAS and SARS-CoV-2 were analyzed. *Results*: Among 4837 GAS tests, 463 (9.6%) were positive. GAS positivity rates varied significantly: 11.4% (pre-COVID-19), 7.1% (restricted social period), and 12.6% (post-restriction period; *p* < 0.001). The proportion of pediatric cases decreased significantly during the restricted social period (24.8–5.3%) before rising sharply in the post-restriction period (47.1%, *p* < 0.001). Among 151 patients tested for GAS and SARS-CoV-2, 14 (9.3%) had co-infections, which were identified exclusively after July 2022. Most patients exhibited mild symptoms, primarily fever and sore throat, with decreased lymphocyte counts despite normal white blood cell counts. *Conclusions*: In our cohort, the incidence of GAS pharyngitis temporarily declined during COVID-19-related public health measures and subsequently increased, particularly among children, after restrictions were lifted. Limited testing may contribute to the underdiagnosis of GAS–SARS-CoV-2 co-infections. Further large-scale studies are warranted to assess microbial interactions, disease severity, and long-term outcomes.

## 1. Introduction

Group A *Streptococcus* (GAS) is a Gram-positive, non-motile bacterium and a common cause of acute pharyngitis, which affects approximately 5–15% of adults and 20–30% of children worldwide [1]. In Japan, GAS pharyngitis (GAS-P) has seasonal outbreaks, which occur from winter to spring and during summer. GAS-P typically presents with sudden fever and sore throat and is often associated with recent contact with infected individuals. In some cases, GAS-P can lead to severe complications, such as pneumonia or bacteremia [1,2]. Additionally, GAS infections can trigger immune-mediated complications, including post-streptococcal glomerulonephritis and rheumatic fever, affecting the kidneys, heart, joints, and other organs [1]. The first-line antimicrobial treatment for GAS-P and the prevention of complications is penicillin-based antibiotics, and macrolides are alternative options [1].

After early 2020, reports from Japan and elsewhere showed a marked decline in GAS-P during widespread non-pharmaceutical interventions (NPI) [3,4,5,6], followed by a resurgence from mid-2022 to 2023 [2,7,8]. However, there are no Japanese studies where adults and children were followed through the entire pre-pandemic, NPI, and post-restriction timeline, leaving it unclear whether the age-specific trends reported nationally are mirrored within a single cohort. Only two single-case reports of GAS-P with concomitant severe acute respiratory syndrome coronavirus 2 (SARS-CoV-2) infection have been published—one during the early (Wuhan/Alpha) wave and one in the Delta wave [9,10]—and we found no peer-reviewed reports describing such co-infections in the Omicron era. The pathophysiology of co-infection and potential microbial interactions between GAS and SARS-CoV-2 remains unclear, partly because of the limited number of reported cases.

Here, we used 6 years of single-center data to examine age-stratified trends in GAS-P and to characterize contemporary GAS–SARS-CoV-2 co-infection.

## 2. Materials and Methods

In this single-center observational study, data from patients who visited Tokyo Shinagawa Hospital (Tokyo, Japan) between 1 January 2019 and 31 December 2024 were retrospectively analyzed. Data on patient age, sex, and GAS and SARS-CoV-2 test results were retrospectively collected from medical records. GAS testing was performed by internal medicine physicians, otolaryngologists, and pediatricians in the outpatient fever clinic and emergency outpatient departments for patients clinically suspected of having pharyngitis. Oropharyngeal swabs were collected by specialized nurses. GAS antigens were detected using Capilia Strep A (TAUNS Laboratories, Inc., Izunokuni, Shizuoka, Japan), which has a sensitivity of 93.1% and a specificity of 100% [11].

We defined two points marking changes in GAS transmission dynamics: May 2020 (when the state of emergency was first declared in Japan) and May 2023 (the time of relaxation of mask mandates, the conclusion of the World Health Organization Public Health Emergency of International Concern, and reclassification of coronavirus disease 2019 (COVID-19) from a Category II-equivalent status to Category V in Japan), based on findings from previous Japanese studies [2,6].

To analyze epidemiological changes, we divided the study period into three phases based on COVID-19-related public health measures: pre-COVID-19 social period (January 2019–April 2020), restricted social period (May 2020–April 2023), and post-restriction period (May 2023–December 2024). We compared patient demographics and clinical characteristics across these periods to evaluate the impact of pandemic-related interventions on GAS transmission and incidence trends.

For statistical analysis, we used the Kruskal–Wallis test to compare age distributions among the three periods, while Fisher’s exact test was applied to compare GAS positivity rates, the sex distribution, and the proportion of pediatric cases. Pairwise comparisons between two periods were subsequently performed using *t*-tests for age and Fisher’s exact test for categorical variables.

All statistical analyses were conducted using R version 4.0.3 (R Core Development Team, Vienna, Austria). Two-sided *p*-values < 0.05 were considered statistically significant. The number of GAS tests performed and positivity rates in our cohort were compared with the incidence of GAS-P reported through the Tokyo Notifiable Disease Surveillance system [6]. Data on the demographic characteristics, symptoms, and clinical characteristics of patients who tested positive for GAS and SARS-CoV-2 simultaneously were collected and processed (Table 1). SARS-CoV-2 was detected using the following methods:

7 May 2020–8 November 2022: in-house polymerase chain reaction (PCR) test;

27 May 2020–31 December 2020: Antigen testing using QuickNavi-COVID-19 Ag (Denka Co., Ltd., Chuo-ku, Tokyo, Japan);

28 December 2020–20 February 2023: ID NOW COVID-19 test (Abbott Laboratories, Abbott Park, IL, USA);

9 November 2022–31 December 2024: GeneXpert SARS-CoV-2 test (Cepheid, Sunnyvale, CA, USA). The criteria for positive results followed contemporary COVID-19 treatment guidelines [12].

**Table 1:** Comparison of GAS-P incidence across different periods.

Variable	Overall	① Pre-COVID-19 Social Period (January 2019–April 2020)	② Restricted Social Period (May 2020–April 2023)	③ Post-Restriction Period (May 2023–December 2024)	*p*-Value (① vs. ②, ② vs. ③, ① vs. ③)
Positivity rate (%)	463/4837 (9.6)	137/1197 (11.4)	169/2398 (7.0)	157/1242 (12.6)	<0.001 (0.000015, <0.001, 0.459)
Age (mean ± SD, years)	28.0 ± 17.2	27.8 ± 16.5	33.1 ± 14.3	22.6 ± 18.9	<0.001 (0.0029, <0.001, 0.013)
Male (%)	278 (60.0)	81 (59.1)	107 (63.3)	90 (57.3)	0.52 (0.48, 0.31, 0.81)
Children aged <15 years (%)	117 (25.3)	34 (24.8)	9 (5.3)	74 (47.1)	<0.001(<0.001, <0.001, 0.00010)

This table presents the positivity rate, age distribution, and demographic characteristics of patients who were positive for GAS across three distinct study periods: the pre-COVID-19 social period (January 2019–April 2020), the restricted social period (May 2020–April 2023), and the post-restriction period (May 2023–December 2024). GAS, group A *Streptococcus*; GAS-P, group A *Streptococcus* pharyngitis; COVID-19, coronavirus disease; SD, standard deviation.

This study was approved by the Ethics Committee of Tokyo Shinagawa Hospital (approval number: 22-A-14) on 25 January 2023. Consent was obtained using the “opt-out method”, thereby allowing patients to decline participation. Therefore, the research ethics committee waived the requirement for written informed consent. Additionally, the graphical abstract and symptom-related icons were generated with the assistance of ChatGPT-4o (OpenAI, May 2024 version), a generative AI tool, and were edited accordingly for presentation purposes.

## 3. Results

**Figure 1:**
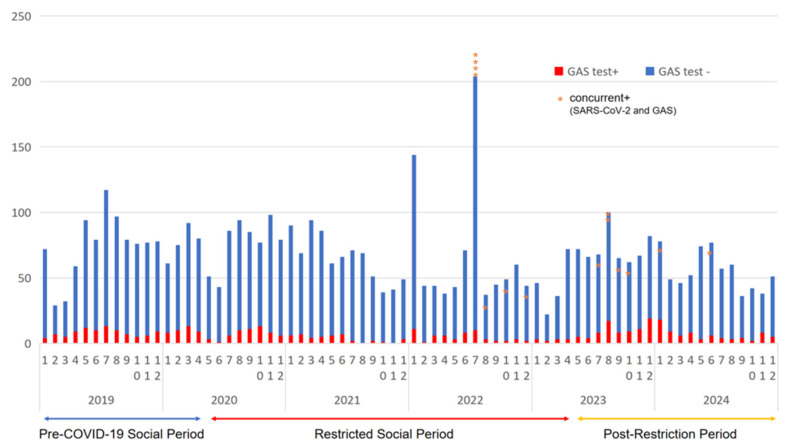
Trends in GAS-P incidence before and after the COVID-19 pandemic. Monthly trends in GAS-P cases between January 2019 and December 2024. Asterisks (*) indicate (number of) cases of concurrent GAS and SARS-CoV-2 infections. GAS-P, group A *Streptococcus* pharyngitis; COVID-19, coronavirus disease 2019; SARS-CoV-2, severe acute respiratory syndrome coronavirus 2.

During the study period, a total of 4837 rapid antigen tests were performed to detect GAS antigens, and 463 (9.6%) were positive for GAS. The number of tests conducted ranged from 22–117 per month, except in January and July 2022. The monthly trends in GAS test results are summarized in Figure 1, while the age and sex distributions of positive cases are presented in Table S1. Among the patients who were GAS-positive, 278 (60.0%) were male, and the overall mean age ± standard deviation (SD) was 28.0 ± 17.2 years (Table 1). In total, 346 (74.7%) of those who were positive for GAS were aged ≥15 years, and 117 (25.3%) were children aged <15 years.

The positivity rate was 11.4% (137/1197) in the pre-COVID-19 social period, significantly decreasing to 7.1% (169/2398, *p* = 0.000015 vs. pre-COVID-19) during the restricted social period, and then increasing to 12.6% (157/1242, *p* < 0.001 vs. restricted, *p* = 0.459 vs. pre-COVID-19) in the post-restriction period. The proportion of children aged <15 years was 24.8% (34/137) in the pre-COVID-19 social period, which significantly decreased to 5.3% (9/169, *p* < 0.001 vs. pre-COVID-19) during the restricted period, then markedly increased to 47.1% (74/157, *p* < 0.001 vs. restricted, *p* = 0.00010 vs. pre-COVID-19) in the post-restriction period.

These findings indicate a significant reduction in GAS positivity rates and pediatric cases during the restricted social period, followed by a sharp resurgence in the post-restriction period, particularly among younger age groups.

Among the 151 patients who tested positive for GAS and underwent simultaneous SARS-CoV-2 testing throughout the study period, 14 were diagnosed with co-infection (Table 2). Their clinical characteristics are shown in Table 3. These co-infection cases spanned a wide age range and included both sexes, with one case occurring in a pediatric patient. All co-infections occurred after July 2022, when the Omicron variant and subsequent lineages predominated.

Despite the co-infection, the clinical course was mild in most cases. Fever was observed in all patients (100%), followed by sore throat (85.7%), cough (50.0%), headache (42.9%), and malaise (42.9%) as the most frequently reported symptoms. None of the patients developed hypoxemia or hypotension, and only three of them required hospitalization because of a severe sore throat, limiting oral intake.

Laboratory findings showed that white blood cell and neutrophil counts remained within normal ranges or were mildly elevated, whereas lymphopenia was common, consistent with known hematologic features of SARS-CoV-2 infections. Pneumonia was detected in only one of the five patients who underwent chest imaging.

All patients responded well to antimicrobial therapy. Penicillin-based antibiotics were most commonly used, followed by macrolides and cephalosporins; approximately one-third of the patients also received antiviral agents. No cases progressed to post-streptococcal complications or long COVID-19.

## 4. Discussion

Our study provides the first contiguous 6-year view of laboratory-confirmed GAS-P in Japan, spanning the pre-COVID-19 era, successive NPI phases, and the post-restriction period. We demonstrated that GAS-P incidence fell sharply after the first state of emergency in May 2020, lost its typical winter–spring and summer peaks, and then rebounded above the pre-pandemic baseline from May 2023—an increase driven predominantly in children.

These phases have been separately addressed in previous studies. The National Institute of Infectious Diseases documented a pandemic-associated collapse of pediatric GAS-P notifications, while an ecological time-series analysis showed a broader decline in communicable diseases during NPIs [13,14].

More recently, surveillance data highlighted a post-2023 surge in GAS-P and streptococcal toxic shock syndrome after public health restrictions were relaxed [15,16]. Using a retrospective enumeration of laboratory-confirmed cases in our center from 2019 to 2024, we linked these separate national observations: our data corroborate the NPI-related suppression of GAS-P, show the documentation of the loss and return of its seasonality and the size quantification of the post-restriction rebound. The initial decrease in the incidence of GAS-P is consistent with observations that stringent NPIs decreased the incidence of other infectious diseases [2,6]. In Japan, the reported incidence of *Streptococcus pyogenes* infections decreased by 32.4% (95% confidence interval: 0.06–0.589) after nationwide mandates on masking, hand hygiene, school closures, and social distancing [2,5,17,18,19]. Because GAS-P peaks are driven largely by child-to-child transmission [19], prolonged masking probably curtailed exposure and delayed immunity accrual [20]. Once mandates were relaxed (13 March 2023 for masks; 8 May 2023 for legal reclassification [21]), incidence rebounded, mirroring the post-restriction surges reported in Europe [22,23,24]. In Japan, the increase lagged until NPIs were lifted despite earlier respiratory syncytial virus and influenza waves [25,26,27], underscoring the dominant effect of behavioral measures on GAS transmission.

A similar study from Italy, in which 2230 swabs were analyzed over a 6-year period (2018–2023) using antigen testing, reported epidemiological trends comparable to those observed in Europe. Despite being a single-center study, a temporary decline in GAS-P cases was documented, followed by a resurgence, aligning with our findings [7].

Nonetheless, in the Italian study, co-infections with viral pathogens were not examined. In our cohort, 14 (9.3%) patients were co-infected with GAS and SARS-CoV-2.

GAS-P symptoms include fever and pharyngodynia, whereas headache, malaise, and cough are COVID-19 symptoms [1,28]. Laboratory findings showed that total leukocyte and neutrophil counts were normal to mildly elevated, although absolute lymphopenia was common (mean lymphocyte count: 0.95 × 10^3^/µL). In contrast, a previous study showed that the prevalence of absolute leukocytosis and relative lymphopenia in GAS-P cases was 78.9% and 56%, respectively; in turn, the prevalence of absolute lymphopenia was less than 10% based on the results of throat cultures [29]. Thus, lymphocyte depletion in our cohort may reflect the additive effect of SARS-CoV-2, which decreases lymphocyte counts even in mild cases [30]. All our patients recovered uneventfully. Nonetheless, the potential for post-streptococcal or post-COVID-19 complications remains unknown and warrants prospective follow-up.

In our cohort, every GAS–SARS-CoV-2 co-infection case was documented after July 2022, a period dominated by Omicron sub-lineages. Notably, these cases emerged while influenza circulation in Japan was minimal, indicating that influenza-related confounding is unlikely. The temporal clustering, therefore, raises the possibility of a positive interaction between the Omicron variant and *S. pyogenes* in the upper airway [27,31]. Since Omicron preferentially replicates in the oropharynx, unlike pre-Omicron strains that infect the lower respiratory tract [32,33,34], two non-mutually exclusive mechanisms are plausible. First, Omicron-mediated epithelial damage may enhance streptococcal adherence and invasion [35,36,37,38]. Second, SARS-CoV-2-induced lymphopenia could impair mucosal immunity; a phenomenon also linked to enhanced *Candida* colonization [39]. Nonetheless, strong evidence for such virus–bacterium synergy is lacking; in our cohort, the high co-infection rate supports this hypothesis but cannot establish causality. Thus, integrated virologic, bacteriologic, and immunologic studies that analyze pathogen loads and mucosal immune profiling are needed to assess whether Omicron facilitates GAS colonization or whether the association reflects the high circulation of both pathogens in community settings.

This study has some limitations. First, SARS-CoV-2 testing among patients who were GAS-positive was not routinely performed, particularly in children, for whom obtaining swab samples poses practical challenges. In addition, the limited testing infrastructure during the early stages of the pandemic may have resulted in the underdetection of co-infections, especially before the Omicron wave, when testing resources were more constrained and diagnostic focus was narrower. Second, no multiplex diagnostic tests capable of detecting *S. pyogenes* and SARS-CoV-2 in a single assay were available in Japan during the study period. Therefore, clinicians ceased testing once one pathogen was identified, potentially missing additional infections [10]. Third, the use of multiple SARS-CoV-2 diagnostic platforms over time, each with varying sensitivities and specificities, may have decreased the accuracy of detection of co-infections. Fourth, the single-center nature of this study may limit the generalizability of the findings. However, the sentinel surveillance of acute respiratory infections nationwide since April 2025, including simultaneous monitoring for GAS and SARS-CoV-2, may help address these knowledge gaps [40,41].

Considering these diagnostic challenges, a substantial number of co-infections with SARS-CoV-2 may have been undetected. Moreover, it is unknown whether these co-infections can be effectively treated with antibiotic monotherapy versus combination therapy with antiviral agents. Thus, SARS-CoV-2 testing should be considered in patients who are GAS-positive with symptoms suggestive of COVID-19, such as cough or lymphopenia.

## 5. Conclusions

During the COVID-19 pandemic, the incidence of GAS-P significantly declined across all age groups, likely because of the widespread implementation of NPIs, which also disrupted the seasonal pattern of GAS-P. The incidence of GAS-P increased after the relaxation of COVID-19 restrictions, particularly among children. Co-infections with GAS and SARS-CoV-2 may be underrecognized owing to limited testing. Moreover, all co-infection cases in our cohort were mild; nevertheless, the potential impact of viral–bacterial interactions on disease severity and long-term complications remains unclear. Thus, larger prospective studies are warranted to elucidate these associations.

## Figures and Tables

**Table 2:** Yearly distribution of GAS positivity and SARS-CoV-2 co-infection among patients.

Category	2019	2020	2021	2022	2023	2024
GAS-positive cases (pediatric cases)	97 (23)	98 (13)	43 (3)	57 (1)	92 (42)	76 (35)
SARS-CoV-2 testing among GAS-positive cases (pediatric cases tested)	0 (0)	21 (0)	5 (0)	42 (1)	43 (3)	40 (5)
Co-infection cases: GAS and SARS-CoV-2 (pediatric co-infections)	0 (0)	0 (0)	0 (0)	7 (0)	6 (1)	1 (0)

This table shows a summary of the yearly counts of patients with GAS-positive results, those tested for SARS-CoV-2, and confirmed co-infections, with pediatric cases shown in parentheses. Co-infection cases were observed only after 2022. GAS, group A *Streptococcus*; SARS-CoV-2, severe acute respiratory syndrome coronavirus 2.

**Table 3:** Clinical characteristics of patients with GAS–SARS-CoV-2 co-infection.

Characteristics	
Total patients	14
Age (mean ± SD)	34.4 ± 17.7
Sex (male/female)	7/7
History of COVID-19	2
Immunodeficiency	0
Number of COVID-19 vaccine doses	
≥2	10
None	4
Days from onset (mean ± SD, [range])	1.5 ± 1.2 [0–4]
Symptoms	
Fever	14 (100%)
Sore throat	12 (85.7%)
Cough	7 (50%)
Headache	6 (42.9%)
Malaise	6 (42.9%)
Arthralgia	4 (28.6%)
Nasal discharge	3 (21.4%)
Muscle pain	2 (14.3%)
Nasal congestion	2 (14.3%)
Chills	2 (14.3%)
Nausea	2 (14.3%)
Vital signs	
Body temperature (mean ± SD, [range])	38.4 ± 1.2 °C [37.1–40.0]
Pulse oximetry oxygen saturation (mean ± SD, [range])	98.0 ± 1.0% [97–99]
Systolic blood pressure (mean ± SD, [range])	122.5 ± 16.9 [90–149]
Heart rate (mean ± SD, [range])	97.1 ± 15.5 [72–120]
Laboratory and imaging data	
White blood cell count (/μL)	5812.5 ± 660.0 [4400–6600]
Neutrophil count (/μL)	4342 ± 911 [3089–5316]
Lymphocyte count (/μL)	954 ± 542 [180–1401]
C-reactive protein (mg/dL)	2.0 ± 1.5 [0.6–4.0]
SARS-CoV-2 PCR (C_t_ values)	20.2 ± 4.3 [15.2–29.4]
Pneumonia findings on chest imaging/patients who underwent imaging (%)	1/5 (20%)
Clinical course and treatment	
Hospitalized/outpatient cases	3/11
Treatment	Regimen Details (Number of Patients)
Antibiotics only (nine patients)	AMPC/CVA (5), CAM (2), ABPC/SBT (1), CFPN-PI (1)
Antibiotics + antiviral agents (five patients)	CCL + ensitrelvir (2), AMPC/CVA + molnupiravir (1), ABPC/SBT + CAM + remdesivir (1), ABPC/SBT + nirmatrelvir (1)
Outcomes	Recovery: 14/14 (100%)

This table shows a summary of the demographic data, symptoms, laboratory findings, and treatment details of 14 patients diagnosed with GAS-P and SARS-CoV-2 infection. ABPC/SBT, ampicillin/sulbactam; AMPC/CVA, amoxicillin/clavulanic acid; CAM, clarithromycin; CCL, cefaclor; CFPN-PI, cefcapene pivoxil; COVID-19, coronavirus disease 2019; GAS, group A *Streptococcus*; GAS-P, group A *Streptococcus* pharyngitis; PCR, polymerase chain reaction; SARS-CoV-2, severe acute respiratory syndrome coronavirus 2; SD, standard deviation.

## Data Availability

The data that support the findings of this study are available from the corresponding author upon reasonable request.

## Supplementary Materials

The following supporting information can be downloaded at https://www.mdpi.com/article/10.3390/medicina61050937/s1: Table S1A: Number of positive cases of group A *Streptococcus* pharyngitis in 2019 and 2020, stratified by sex and age group; Table S1B: Number of positive cases of group A *Streptococcus* pharyngitis in 2021 and 2022, stratified by sex and age group; Table S1C: Number of positive cases of group A *Streptococcus* pharyngitis in 2023 and 2024, stratified by sex and age group.

## Abbreviations

The following abbreviations are used in this manuscript:

COVID-19	coronavirus disease 2019
GAS	group A *Streptococcus*
GAS-P	group A *Streptococcus* pharyngitis
NPIs	non-pharmaceutical interventions
PCR	polymerase chain reaction
RSV	respiratory syncytial virus
SARS-CoV-2	severe acute respiratory syndrome coronavirus 2
SD	standard deviation
