# Supplementary material for: Trends in Group A Streptococcus Pharyngitis and Co-Infection with Severe Acute Respiratory Syndrome Coronavirus 2: A Retrospective Observational Study
Source: Medicina (Kaunas). 2025 May 21;61(5):937. doi: 10.3390/medicina61050937 (PMC12113336; doi:10.3390/medicina61050937)
Supplement: Supplementary file 1 [file medicina-61-00937-s001.zip › medicina-3583097-supplementary.pdf]

Table S1A. Number of positive cases of group A *Streptococcus* pharyngitis in 2019 and 2020 stratified by sex and age group

	2019												2020											
	**Jan**	**Feb**	**Mar**	**Apr**	**May**	**Jun**	**Jul**	**Aug**	**Sep**	**Oct**	**Nov**	**Dec**	**Jan**	**Feb**	**Mar**	**Apr**	**May**	**Jun**	**Jul**	**Aug**	**Sep**	**Oct**	**Nov**	**Dec**
**Total number of patients**	4	7	5	9	12	10	13	10	7	5	6	9	8	10	13	9	3	1	6	10	11	13	8	6
**Male:female**	1:3	5:2	2:3	5:4	10:2	3:7	9:4	7:3	4:3	3:2	3:3	8:1	6:2	5:5	6:7	4:5	1:2	1:0	3:3	6:4	8:3	10:3	6:2	1:5
**Age group, years**																								
0–4		2		2	2	2			1	1	2	2	2	1	2	1						1		
5–9						3	1	1				1		1	1	2						1		
10–14						1		1				1		1										
15–19					1																	1	1	
20–29	2	3	3	4	3	2	4	3	1	1	3			3	5		1	1	2	3	5	5	4	4
30–39	1		1	2	2	1	4	3	4	2		3	6	3	4	3	1		2	3	2	3		1
40–49		2	1		2	1	4	1			1				1	2			2	1	2	2	2	
50–59				1	2			1						1		1				1	2			1
60–69	1									1		1								1			1	
70–79									1			1					1							
80–89																				1				

Table S1B. Number of positive cases of group A *Streptococcus* pharyngitis in 2021 and 2022 stratified by sex and age group

	2021												2022											
	**Jan**	**Feb**	**Mar**	**Apr**	**May**	**Jun**	**Jul**	**Aug**	**Sep**	**Oct**	**Nov**	**Dec**	**Jan**	**Feb**	**Mar**	**Apr**	**May**	**Jun**	**Jul**	**Aug**	**Sep**	**Oct**	**Nov**	**Dec**
**Total number of patients**	6	7	4	5	6	7	2	0	2	1	0	3	11	1	6	6	3	8	10	3	2	2	3	2
**Male:female**	5:1	4:3	2:2	5:0	5:1	2:5	2:0		1:1	0:1		1:2	7:4	1:0	5:1	6:0	3:0	2:6	5:5	2:1	2:0	2:0	3:0	1:1
**Age group, years**																								
0–4	1			1								1											1	
5–9																								
10–14																								
15–19		5										1										1*		
20–29	3	2	3		2	5							4	1	2	2	2	4	3*	2		1		2*
30–39				1	3	1	1		1	1		1	4		2	4	1	1	3**	1*	1			
40–49				2		1	1		1				2		2			1	3					
50–59	2		1										1					1					1	
60–69				1															1*				1	
70–79					1													1			1			
80–89																								

*Cases positive for *Streptococcus* and severe acute respiratory syndrome coronavirus 2.

Table S1C. Number of positive cases of group A *Streptococcus* pharyngitis in 2023 and 2024 stratified by sex and age group

	2023												2024											
	**Jan**	**Feb**	**Mar**	**Apr**	**May**	**Jun**	**Jul**	**Aug**	**Sep**	**Oct**	**Nov**	**Dec**	**Jan**	**Feb**	**Mar**	**Apr**	**May**	**Jun**	**Jul**	**Aug**	**Sep**	**Oct**	**Nov**	**Dec**
**Total number of patients**	3	2	3	3	5	4	8	17	8	9	11	19	18	9	6	8	3	6	4	3	4	2	8	5
**Male:female**	1:2	1:1	2:1	1:2	2:3	3:1	5:3	9:8	6:2	5:4	4:7	9:10	11:7	5:4	3:3	5:3	2:1	4:2	1:3	2:1	2:2	2:0	6:2	4:1
**Age group, years**																								
0–4		1			1	1	1	3	1	1	1	5	3	3	1	2	1		1	1			1	1
5–9				1	1		2	2*	5	5	4	6	5	2	4			1			1	1	4	1
10–14	1																	1						1
15–19													1					1						
20–29	1		1	1	1		3	5	1*	1	2	2	1		1	1		2*	1	1		1		1
30–39		1	1	1	1	2		5*	1		1	3	3	1		1	1	1	1		2		1	
40–49						1	2*	1		1	2	2	3	2		2			1	1			1	1
50–59	1		1								1	1	1*	1			1				1			
60–69					1			1		1*			1			1								
70–79																							1	
80–89																1								

*Cases positive for *Streptococcus* and severe acute respiratory syndrome coronavirus 2.
